# Heterogeneity of Skin Surface Oxygen Level of Wrist in Relation to Acupuncture Point

**DOI:** 10.1155/2012/106762

**Published:** 2012-05-16

**Authors:** Minyoung Hong, Sarah S. Park, Yejin Ha, Jaegeun Lee, Kwangsik Yoo, Gil-Ja Jhon, Minah Suh, Youngmi Lee

**Affiliations:** ^1^Department of Biological Science, Sungkyunkwan University, Suwon 440-746, Republic of Korea; ^2^Department of Chemistry and Nano Science, Ewha Womans University, Seoul 120-750, Republic of Korea; ^3^Graduate Program for Health Science and Technology, Sungkyunkwan University, Suwon 440-746, Republic of Korea

## Abstract

The distribution of partial oxygen pressure (pO_2_) is analyzed for the anterior aspect of the left wrist with an amperometric oxygen microsensor composed of a small planar Pt disk-sensing area (diameter = 25 **μ**m). The pO_2_ levels vary depending on the measurement location over the wrist skin, and they are systematically monitored in the analysis for both one-dimensional single line (along the wrist transverse crease) and two-dimensional square area of the wrist region. Relatively higher pO_2_ values are observed at certain area in close proximity to the position of acupuncture points with statistical significance, indicating strong relationship between oxygen and acupuncture point. The used oxygen microsensor is sensitive enough to detect the pO_2_ variation depending on the location. This study may provide information helpful to understand possible physiological roles of the acupuncture points.

## 1. Introduction

Acupuncture is a method of medical treatments, based on inserting small needles on the specified body skin locations called acupuncture points. The practice of acupuncture as a healing treatment dates back over 2500 years in traditional eastern medicine. In the late 20th century, acupuncture became to be accepted as an alternative and complementary therapy even in western countries including the United States [1]. In fact, the National Institute of Health (NIH) published a consensus on the use acupuncture in the treatment of pain symptom in 1997 [2]. 

According to acupuncture meridian theory, a network of 12 main meridians passes through body internal organs and links acupuncture points on skin together. Through the meridian channels, a vital energy, called Qi, circulates the body to regulate body functions. Acupuncture is considered to stimulate the Qi circulation to attain the balance of Qi. Although acupuncture practice is widely used for chronic illness, the efficacy and mechanism of the acupuncture action in mediating analgesia still remain controversial. Indeed, the lack of anatomical and scientific evidence supporting the existence of meridians, acupuncture points, and Qi makes more difficult for the acupuncture treatment to be generally accepted in modern science. Some research efforts for anatomical studies of meridians and acupuncture points have been reported [3–11]. However, there is still controversy since the experimental results could not provide direct/obvious evidence, and moreover they often show a lack of reproducibility.

In acupuncture studies, three kinds of Qi are described to be obtained from air, food, and inheritance, suggesting the close relationship between Qi and air (i.e., oxygen) [1]. Oxygen is essential for energy metabolism in most living organisms. Meanwhile, higher expression of nitric oxide (NO) synthase enzyme, producing endogenous NO, was reported around skin acupuncture points and meridians than other areas [12]. NO is a well-known vasodilator increasing blood flow and volume and therefore relates to oxygen transport in body [13]. From these separate reports, we inferred that acupuncture points were possibly associated with body oxygen supply and, therefore, recently reported the real-time quantitative measurements of oxygen levels on acupuncture points using a highly sensitive electrochemical oxygen microsensor [14]. The localized oxygen levels at small acupuncture points and at nearby nonacupuncture points were measured successfully, because of the small planar dimension of the sensor (sensing diameter = 25 *μ*m). In fact, the oxygen levels measured at two acupuncture points (LI4 (Hegu) and PC8 (Laogong)) were observed to be higher than those at the corresponding nonacupuncture points, providing an evidence of the physical existence of acupuncture points which may be functionally connected with the oxygen supply [14]. Advanced from the previous work, this paper reports the blind measurements of oxygen levels within confined areas of wrist skin surface and the relationship between the oxygen levels with acupuncture points.

## 2. Materials and Methods

### 2.1. Electrochemical Oxygen Microsensor

A clark-type amperometric microsensor for selective oxygen measurement was fabricated as described previously [14]. The oxygen microsensor consists of a glass-sealed Pt disk cathode (Pt diameter = 25 *μ*m, Good Fellow) and a coiled Ag/AgCl wire anode (127-*μ*m diameter, A-M Systems) covered with PTFE gas-permeable membrane (W. L. Gore & Associates, thickness < 19 *μ*m, porosity 50%, pore size 0.05 *μ*m). The composition of an internal solution, both the cathode and anode, is 30 mM NaCl and 0.3 mM HCl in deionized water. The surface of the Pt disk cathode was electrochemically platinized using platinizing solution (YSI Inc., Yellow Springs, OH) to increase the real active surface of the electrode and eventually to enhance the sensor sensitivity to oxygen [15]. A potential of −0.6 V (versus Ag/AgCl anode) was applied to the Pt cathode where the electrochemical reduction of oxygen occurs favorably at this potential. The current between the cathode and anode, induced by the oxygen reduction, was monitored as a function of time using CHI1000A electrochemical analyzer (CH Instruments Inc., USA). As-prepared oxygen microsensor was calibrated before and after oxygen measurements by recording the sensor current at −0.6 V with successive several injections of a given amount of phosphate-buffered saline (PBS, pH 7.4, Fisher Scientific) solution saturated with oxygen into deaerated PBS (pH 7.4) solution to alter the oxygen concentrations.

### 2.2. Oxygen Measurements on Wrist Skin

The experimental details for the measurements of oxygen levels on skin are described previously [14]. Briefly, the prepared oxygen microsensor was positioned above the first wrist skin point of interest, which was wetted with a drop of PBS (pH = 7.4) solution (15 *μ*L). A micromanipulator (World Precision Instrumentation Inc., Sarasota, FL, USA) was used to position the sensor and maintain the separation between the sensor and skin surface, ~1 mm. Then, the sensor current between the cathode and anode, which is proportional to the partial oxygen pressure (pO_2_), was recorded using an electrochemical analyzer. Once the measured current reached to a quite stable one, the sensor was moved horizontally to the second skin point of interest while the sensor current was monitored continuously. After the stable current was achieved at the second point, the sensor was moved to the third point to measure the pO_2_ level at that location. This whole procedure was repeated until the measurements of pO_2_ levels for all the projected points were finished.

**Figure 1:**
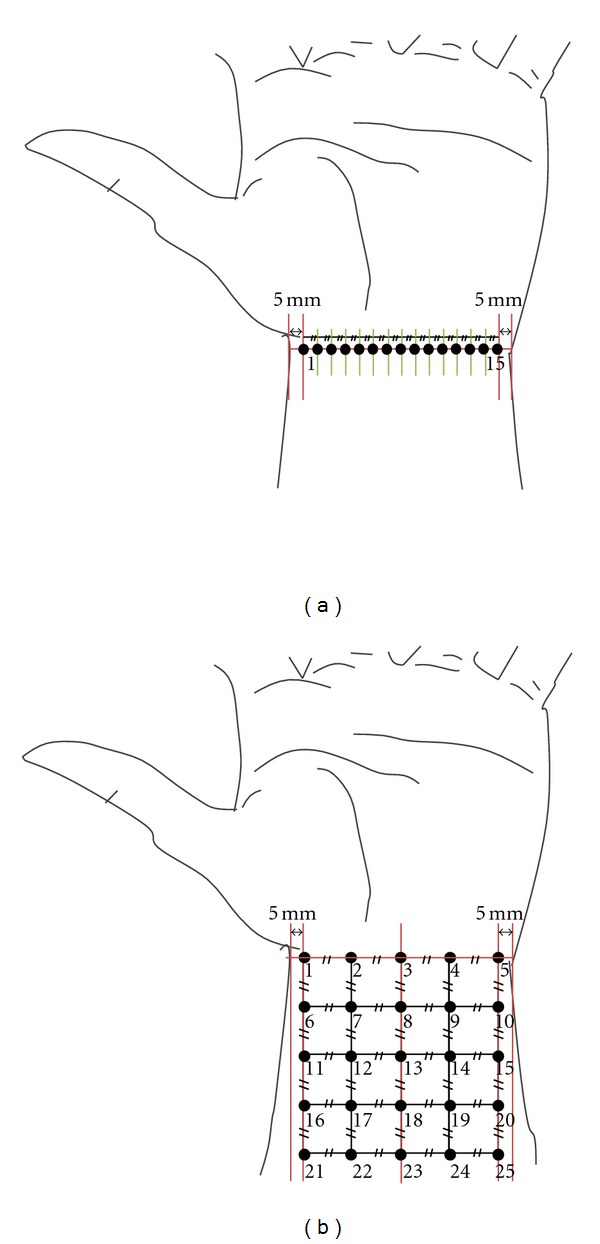
Schematic illustration for the points on the wrist skin where an oxygen microsensor was positioned for pO_2_ analysis: (a) one-dimensional and (b) two-dimensional measurement. The points, No. 1, 15 in (a) and the points, No. 1, 5 in (b) were positioned 5 mm apart from the left and right sides of the wrist. Symbol, //, represents the same separation.

The measurements of pO_2_ levels were performed for (1) one-dimensional single line and (2) two-dimensional square area within the wrist independently. For the one-dimensional experiment, the sensor currents responding to pO_2_ levels were measured at 15 different points along the lateral line on the anterior aspect of the left hand-wrist transverse crease. The 15 points were evenly distributed with the same separation (*d* = 3–3.5 mm depending on individual subject) between two adjacent points along the transverse wrist crease line. The first point and the last 15th point were positioned 5 mm apart from the left and right sides of the wrist as shown in Figure 1(a).

For the two-dimensional analysis, the first 5 points were evenly positioned with the same separation between two adjacent points (*d* = 10–12 mm depending on individual subject) along the lateral line on the anterior aspect of the left hand-wrist boundary crease. Again, the first point and the fifth point were positioned 5 mm apart from the left and right sides, respectively. The central five points (nos. 3, 8, 13, 18, and 23) were positioned along the centered vertical line dividing the anterior wrist evenly, with the same separation (*d*) as the one for the first lateral wrist line. Then, the other points could be distributed while keeping the same point-to-point separation as shown in Figure 1(b).

The measurements were carried out for five healthy volunteers (average age = 24.2) in calm and rest conditions at room temperature. None of the subjects were previously treated with acupuncture needle insertion at the skin locations investigated. The measured sensor currents were converted to the corresponding pO_2_ levels using prior calibration data.

### 2.3. Data Analysis and Statistics

For each volunteer subject, the pO_2_ levels measured twice and these two pO_2_ values obtained at the same location were averaged, and the standard deviation was also calculated independently. The averaged data obtained at the same skin location of five different subjects were also averaged. The data for a few specific points showing relatively higher pO_2_ values than the other region were compared with that at other points exhibiting relatively lower pO_2_ values using two tailed *t*-test with a Bonferroni correction. *P* value < 0.05 was considered significantly different in statistical meaning.

## 3. Results and Discussion

**Figure 2:**
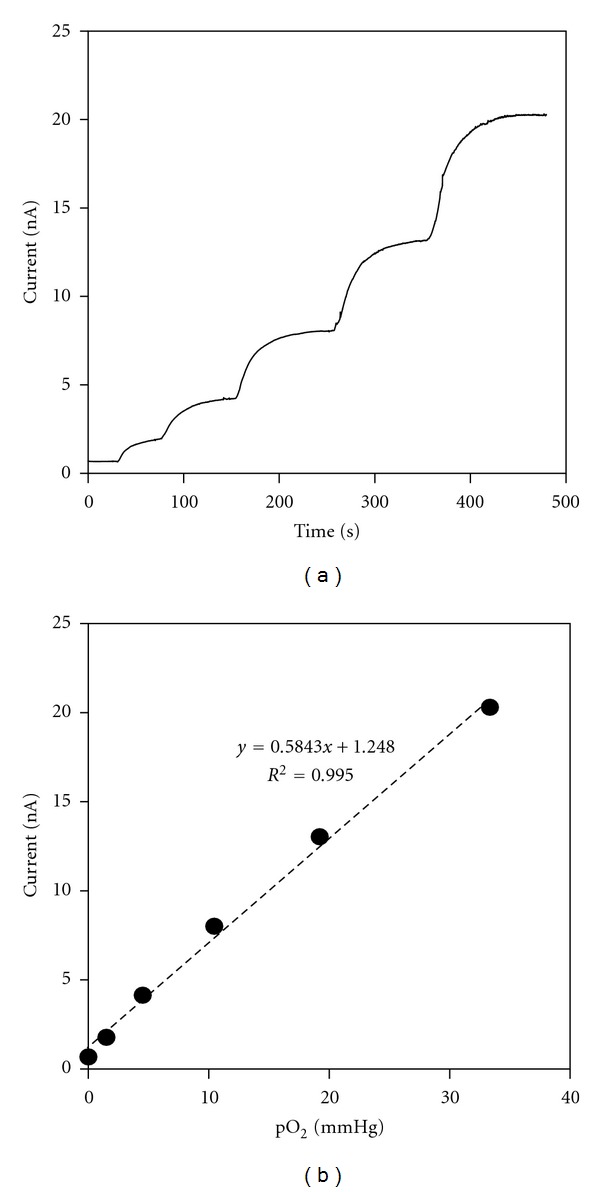
(a) A typical current response curve of an oxygen microsensor with the varied oxygen concentration. (b) Corresponding calibration curve in terms of pO_2_.

The analytical performance of an amperometric oxygen microsensor was characterized.  Figure 2(a) shows the dynamic sensor response obtained by measuring the sensor current responding to the pO_2_ value which was altered by successive injections of a given amount of oxygen standard solution into a deareated PBS sample solution. The sensor current increases in proportion to pO_2_ value, and the corresponding calibration curve (Figure 2(b)) shows reasonable linearity and sensitivity of 523.8 ± 58.0 pA/mmHg (*n* = 7). The sensor sensitivity varied within < ~2% before and after the skin oxygen measurements and <0.5% for 10°C temperature change (25–35°C), confirming the sensor stability.

**Figure 3:**
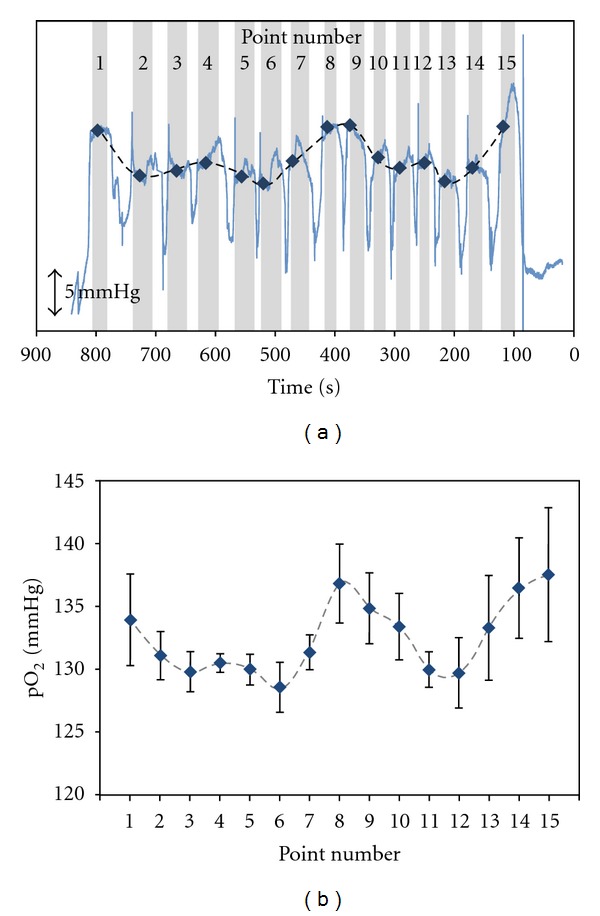
(a) A representative pO_2_ measurement along the wrist transverse crease as shown in Figure 1(a). The pO_2_ values were measured continuously at 15 different points with the one-dimensional sensor movement. Note that time increases to the left direction in *x* axis, since the measurement was carried out with the sensor movement from point no. 15 to point no. 1. The scattered symbols representing the pO_2_ values measured at corresponding points are overlaid for clear presentation. (b) Averaged pO_2_ levels (*n* = 5) for 15 different points. The sensor's measurements at each point were averaged across five subjects. A paired *t-*test with a Bonferroni correction was conducted for six representative points (nos. 4, 6, 8, 10, 14, and 15), and the *P* values are summarized in Table 1.

Figure 3 shows typical data showing the pO_2_ values monitored at 15 different locations along the transverse wrist crease line. The sensor current measured in terms of time was converted to the pO_2_ value based on the prior sensor calibration data. Gray-colored sections represent the measurements made over the projected points with the sensor-to-skin separation of ~1 mm. Current signals observed in noncolored sections are induced by the sensor movement from one to the other points. In fact, the comparatively higher pO_2_ values than the other regions are observed at the regions around the points, nos. 1, 8, 9, and 15. For all five different subjects without exception, similar patterns to Figure 3(a) were observed.  Figure 3(b) displays the pO_2_ value averaged for five entire subjects (with standard deviation) corresponding to each point. The pO_2_ at each point was taken as the average for the data obtained for the last 60 s of the overall measurement time at that point before the sensor movement to another point. This makes sure that the fully equilibrated pO_2_ value is obtained. Interestingly, the pO_2_ values are observed to be closely related to the positions of acupuncture points. In fact, three acupuncture points, LU9 (Taiyuan), PC7 (Daling), and HT7 (Shenmen) from left to right side, are known along the transverse wrist crease line. Rather large standard deviations of the averaged pO_2_ values could be ascribed to the interindividual variation such as wrist circumference. In the one-dimensional study, the measured pO_2_ value was in the range of 125–143 mmHg with the greatest difference between the highest and lowest pO_2_ that was 5.7–9.2 mmHg depending on the individual subject.

In addition, the pO_2_ levels at six representative points (nos. 5, 6, 8, 11, 14, and 15) were compared with one another. A two-tailed *t-*test with a Bonferroni correction verifies that the relatively higher pO_2_ values at the points, nos. 8, 14, and 15 are significantly different from the lower pO_2_ values at the points, nos. 5, 6, and 11 (Table 1). Current study is in good agreement with our previous work reporting higher pO_2_ levels at the acupuncture points (LI4 and PC8) than at nonacupuncture points.

As indicated in the Methods section, the pO_2_ analysis was also carried out at 25 different points evenly distributed in two-dimensional square area over the wrist as depicted in Figure 1(b). The measured pO_2_ values showed even higher interindividual variation in this two-dimensional analysis compared to the one-dimensional one. It is presumably considered that the subject body size difference induces a relatively large variance in the analysis of a wider region. Therefore, typical measurement examples are presented without the statistical analysis of the overall subjects. 

For convenient comparison purpose, each measured pO_2_ was normalized to the average of all 25 values. In fact, the normalized pO_2_ was obtained as follows:

(1) $$\;{\text{pO}}_{2,\mathrm{norm}}=\frac{{\text{pO}}_{2}}{{\text{pO}}_{2,\text{avg}}},$$

where pO_2,norm⁡_ is the normalized pO_2_; pO_2_ is the measured pO_2_ value at each point; pO_2,avg_ is the average of all the pO_2_ values measured at 25 different point for each subject.

Thus, the points exhibiting the pO_2,norm⁡_ values less or greater than 1 represent the areas where the measured pO_2_ is lower or higher than the average, respectively. Each pO_2,norm⁡_ was obtained by averaging the two pO_2,norm⁡_ values obtained from two separate measurements at the corresponding same location for each subject. The average values of 25 pO_2_ values were laid within a range of 130–140 mmHg for five subjects.

**Figure 4:**
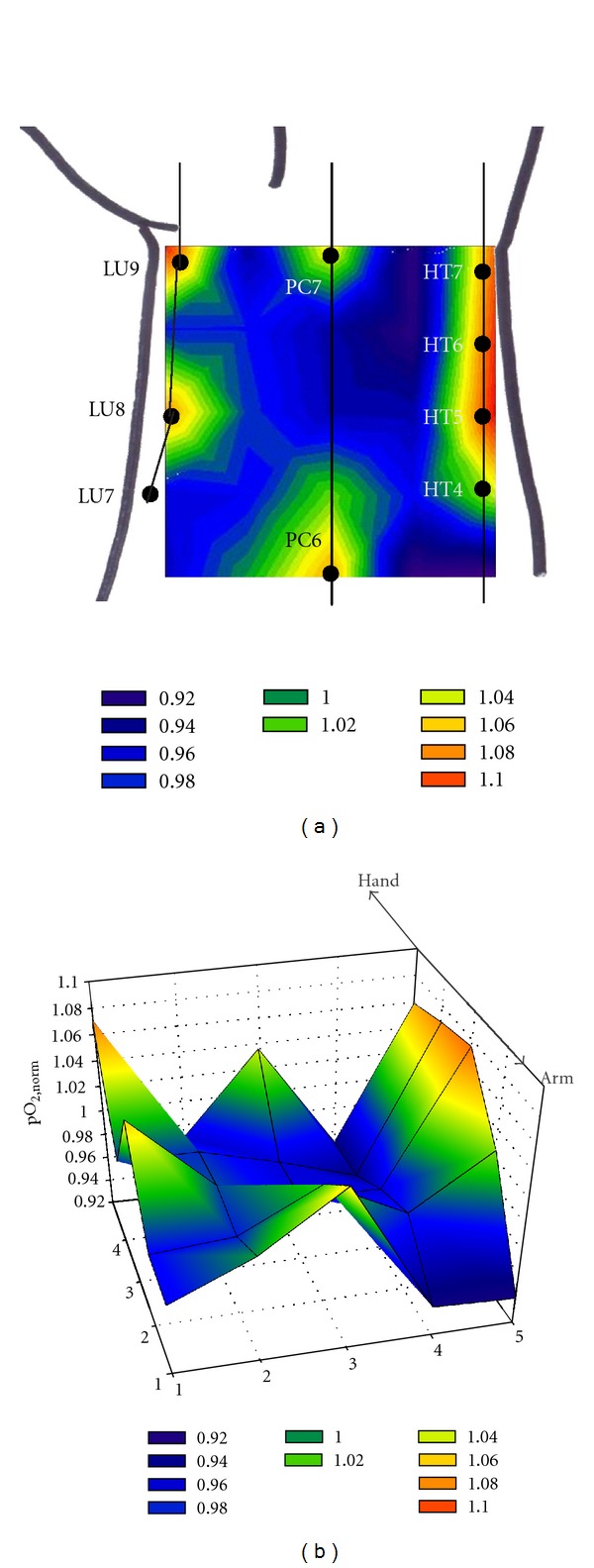
(a) 2-D and (b) 3-D illustration for the color-coded contour plots for a typical example of the two-dimensional oxygen measurement over the wrist skin. A linear change in the pO_2,norm⁡_ values was assumed between two adjacent points.

**Figure 5:**
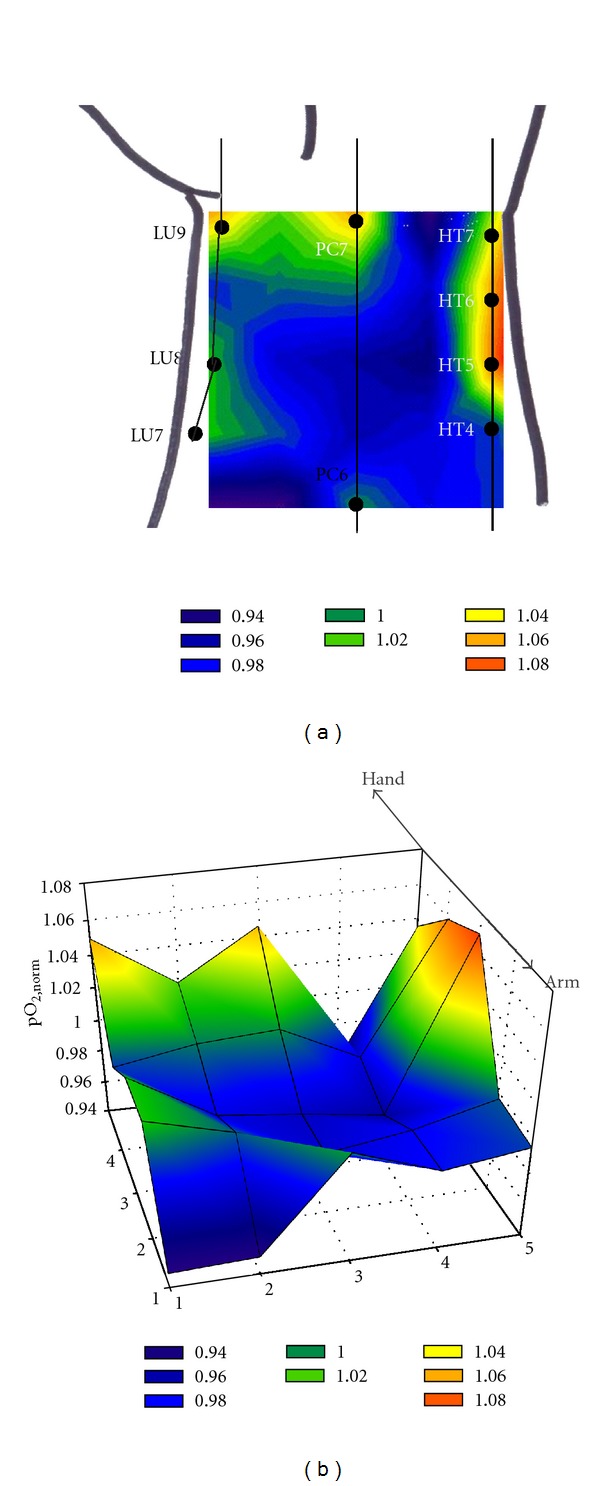
(a) 2-D and (b) 3-D illustration for the color-coded contour plots for another typical example of the two-dimensional oxygen measurement over the wrist skin. A linear change in the pO_2,norm⁡_ values was assumed between two adjacent points.

Figure 4 is the color-coded contour plots of a typical two-dimensional oxygen measurement for one subject. For these contour plots, a linear change in pO_2_ was assumed between two adjacent points. As in the one-dimensional experiment, the pO_2_ values were varied depending on the locations, showing the heterogeneity of skin oxygen levels. The acupuncture point-high pO_2_ relationship is observed more clearly in the two-dimensional analysis. According to Eastern medicine, there are eight acupuncture points known in the wrist region for which the oxygen measurements were carried out: LU9 (Taiyuan) and LU8 (Jingqu) on the lung meridian; PC7 (Daling) and PC6 (Neiguan) on the pericardium meridian; and HT7 (Shenmen), HT6 (Yinxi), HT5 (Tongli), and HT4 (Lingdao) on the heart meridian. The locations of these acupuncture points are indicated in Figure 4(a). In fact, the pO_2,norm⁡_ values are greater than 1 (i.e., higher pO_2_ level than the average) at the points close to the area where these acupuncture points are supposed to be present. Another typical example is shown in Figure 5. In this example, heterogeneous pO_2_ distribution over the wrist is also observed, obviously showing the strong correlation between the acupuncture point and high pO_2_ level. Similar pO_2_ distribution patterns were observed for overall five subjects. 

As indicated in our previous report [14], the measured pO_2_ value shows a dependence on the distance between the sensor end plane and skin. In fact, the higher pO_2_ was observed when the sensor-skin separation was shorter. However, the pO_2_ variation induced by a slight difference in the sensor-to-skin separation during the experimental course was relatively small. In fact, pO_2_ variation < 1 mmHg was observed from 10 separate measurements at the same point of one subject, supporting the reliability of the sensor movement/reposition procedure. Since the pO_2_ difference depending on the location is reasonably greater than the one induced by the sensor vertical positioning, the observed heterogeneous pO_2_ distribution can be considered to be valid.

The reason for the relatively higher pO_2_ levels around acupuncture points is not clearly understood yet. One possible explanation is that the oxygen supply by capillary oxygen transport is greater, and the oxygen uptake from the atmosphere is lesser around the acupuncture points than the other area. The oxygen supply to skin was demonstrated as a balance between the oxygen transport by blood and uptake from the atmosphere by Stücker et al. [16, 17]. Therefore, large blood vessels or Primo-nodes/vessels would possibly exist underneath the skin acupuncture points. Primo-vascular system was first proposed to be corresponding to acupuncture points by Kim [18] and recently rediscovered as a new circulatory system by Seoul National University group [19]. Further research, such as pO_2_ analysis combined with anatomical study, needs to be performed to clarify the possible relationship between Primo-vascular system and acupuncture points.

Regarding the connection between gas and acupuncture points, higher pO_2_ at the acupuncture points was previously reported for rabbits while the pO_2_ was measured in the tissue ~0.5 cm below four selected skin acupuncture points (ST36, ST37, CV16, and CV17) with the sensor insertion [20]. In addition, higher transcutaneous carbon dioxide emission at 12 acupuncture points on the pericardium meridian was reported compared with control points beside the meridian line [21]. Including these reports, the research regarding the acupuncture points and meridian has been conducted by comparing the characteristics of acupuncture points with that of control. Our current work provides clearer evidence on the strong pO_2_-acupuncture point correlation successfully by extending the analysis to the two-dimensional as well as noninvasive one. This investigation may verify the physical existence of the acupuncture points and provide helpful information to understand their physiological/biological functions believed in Eastern medicine for ages.

## 4. Conclusions

The skin surface pO_2_ levels measured for the anterior aspect of the left wrist varied depending on the locations in both the one-dimensional and two-dimensional analyses. The regions showing relatively higher pO_2_ levels compared to the other regions showed a strong correlation to the positions of acupuncture points for entire five subjects without exception. The higher pO_2_ values near the acupuncture points were observed with statistical significance. The used amperometric oxygen microsensor to monitor pO_2_ provided high sensitivity and small disk sensing area (diameter = 25 *μ*m) which are sufficient to detect the pO_2_ variation as a function of location. Current study provides direct and scientific evidence on the physical existence of acupuncture points and may contribute to understand their possible biological/physiological functions.

## Figures and Tables

**Table 1:** Calculated *P* values for the paired *t*-test (*P < 0.05).

Point no.	P6	P8	P11	P14	P15
P5	0.0561	0.0181*	0.962	0.0369*	0.0488*
P6		0.0157*	0.361	0.0267*	0.0403*
P8			0.0015*	0.856	0.677
P11				0.0165*	0.0149*
P14					0.499
